# Regulation of AMPK-related glycolipid metabolism imbalances redox homeostasis and inhibits anchorage independent growth in human breast cancer cells

**DOI:** 10.1016/j.redox.2018.04.016

**Published:** 2018-04-18

**Authors:** Lin Yang, Zihao He, Jingyue Yao, Renxiang Tan, Yejin Zhu, Zhiyu Li, Qinglong Guo, Libin Wei

**Affiliations:** a State Key Laboratory of Natural Medicines, Jiangsu Key Laboratory of Carcinogenesis and Intervention, China Pharmaceutical University, 24 Tongjiaxiang, Nanjing 210009, People's Republic of China; b State Key Laboratory Cultivation Base for TCM Quality and Efficacy, Nanjing University of Chinese Medicine, 138 Xinlin Road, Nanjing 210023, People's Republic of China

**Keywords:** Redox homeostasis, Pentose phosphate pathway, Fatty acid oxidation, Anti-metastasis, GL-V9

## Abstract

Breast cancer is one of the most lethal tumors in the world, among which 15% are triple-negative breast cancers (TNBCs) with higher metastasis and lower survival rate. Anoikis resistance is a key process during tumor metastasis, which is usually accompanied with metabolism reprogram. In this study, we established an anchorage independent growth model for MDA-MB-231 cells and investigated the changes in metabolism and redox homeostasis. Results showed that during detached-growth, MDA-MB-231 cells tend to generate ATP through fatty acid oxidation (FAO), instead of glycolysis. Amount of glucose was used for pentose phosphate pathway (PPP) to keep redox balance. Moreover, we discovered that a synthesized flavonoid derivative GL-V9, exhibited a potent inhibitory effect on the anchorage independent growth of TNBCs in vitro and anti-metastasis effect in vivo. In terms of the mechanism, GL-V9 could promote the expression and activity of AMPK, leading to the decrease of G6PD and the increase of p-ACC. Thus, the level of PPP was suppressed, whereas FAO was highly enhanced. The reprogram of glycolipid metabolism destroyed the redox balance ultimately and induced cell death. This paper indicated a novel regulating mechanism of redox homeostasis involving with glycolipid metabolism, and provided a potential candidate for the anti-metastatic therapy of TNBCs.

## Highlights

• Instead of glycolysis, FAO is the dominant way for ATP generation in anchorage independent growth.
• Glucose in cells detached from EMC is used for PPP to resist the ROS form OXPHOS.
• GL-V9 inhibits anchorage independent growth via imbalancing the redox homeostasis.
• AMPK is the critical regulator in GL-V9-induced glycolipid metabolism reprogram.

## 1 Introduction

Breast cancer is one of the most lethal tumors in the world. In the United States, breast cancer is the most commonly diagnosed cancer among women excluding skin cancers and is the second cause of cancer death after lung cancer. In 2017, the number of new cases and breast cancer deaths reached 252,710 and 40,610 respectively [1]. In China, breast cancer is the most common cancer among female, with the incidence 17.07% and 278,800 new cases, ranking fifth in the causes of tumor death after cancers of lung, gastric, liver and colorectum [2]. Among all the breast cancer cases, 15% are triple-negative breast cancers (TNBCs), which lack expression of estrogen receptor (ER), progesterone receptor (PR), and human epidermal growth factor receptor 2 (HER2) and have a very aggressive disease course [3]. 10–20% of women who have TNBC subtype breast cancers usually have shorter survival due to high malignancy, high recurrence rate and high transferability [4]. 1–3 years after TNBCs are diagnosed, tumors can easily transfer to internal organs and 40% of the metastasis occurs in lungs [5].

Metastasis to distant sites is a substantial barrier in cancer therapy and may cause 90% of human cancer deaths [6], [7], [8]. During the distant metastasis, cancer cells need to travel through blood vessels or lymphatic vessels after they leave the primary lesions. Normal epithelial cells depend on the adhesion to the extra-cellular matrix (ECM) for survival, proliferation and differentiation [9]. Once detached from the ECM, caspase-mediated apoptosis may be activated, which is known as anoikis [10]. However, during tumor metastasis, cancer cells must adapt to the condition of detachment from ECM while they are traveling around the circulatory system. This kind of growth is also known as anchorage-independent growth [11], [12], [13]. In the progress of anchorage-independent growth, a distinct variety of cellular and molecular alterations may contribute to the viability of cancer cells, indicating that cancer cells own their own regulation of anoikis resistance [9].

An alternative route of anoikis inhibition is high levels of reactive oxygen species (ROS), which can activate SRC pathway [14]. ROS-mediated activation of SRC contributes to anoikis inhibition through ERK-mediated modulation of BIM-EL [15], [16], [17]. However, a substantial reduction in glucose uptake and ATP was observed after MCF-10A cells were cultured in non-adherent dishes [18]. Researches showed that in unanchored breast cancer cells, the contribution of fatty acid oxidation (FAO) for ATP production was extremely enhanced, no matter the glucose was deprived or not [18], [19]. Under this condition, fatty acid, instead of glucose, became the main resource of oxidative phosphorylation (OXPHOS) and increased ROS level. Meantime, the glucose metabolism in oxidative branch of pentose phosphate pathway (PPP) was highly activated, which produced amount of NADPH and kept the balance of redox status. Thus, the balance of glycolipid metabolism plays a vital role in anchorage-independent growth. Once the balance is broken, the high level of ROS would be toxicity for the cancer cells under anchorage-independent growth.

One of the hallmarks of cancer is reprogramming of energy metabolism, among which an anomalous character regarded as ‘Warburg effect’ is aerobic glycolysis [7]. The deregulating metabolism has been proven to be related to tumor metastasis. Under hypoxia conditions, both a switch to glycolysis and the acid microenvironment promote expressions of angiogenetic factors which ultimately enhance tumor metastasis [20]. In addition, the consumption of glucose produces some by-products, such as lactic acid etc., which meet the needs of cancer metastasis [21]. PPP is generally associated with metastasizing cancers [22], which not only provides ribose of nucleotides production, but also generates NADPH for macromolecular synthesis and ROS scavenger [23]. Thus, targeting metabolism of tumor cells may be an effective way in tumor metastasis therapy.

In the present report, we investigated the anticancer mechanism in breast cancer of a newly synthesized flavonoid GL-V9 (5-hydroxy-8-methoxy-7-(4-(pyrrolidin-1-yl) butoxy)− 4H-chromen-4-one). Previous studies demonstrated that GL-V9 inhibited tumor invasion via down-regulating the expression and activity of matrix metalloproteinase-2/9 in MDA-MB-231 and MCF-7 cell in vitro [24]. However, whether and how GL-V9 influenced the process of metastasis in vivo was still unknown. Herein, based on the metabolism reprogram of breast cells in anchorage-independent growth, we investigated the mechanism that GL-V9 inhibited the metastasis involving anoikis resistance both in vitro and in vivo. According to our findings, GL-V9 could inhibit anchorage-independent growth and metastasis of human breast cancer cells through the regulation of AMPK-related glycolipid metabolism.

## 2 Materials and methods

### 2.1 Reagents

GL-V9 (C_24_H_27_NO_5_,(5-hydroxy-8-methoxy-2-phenyl-7-(4-(pyrrolidin-1-yl) butoxy)4H-chromen-4-one), MW 409.47, purity 99%) is a new flavonoid derivative and synthesize by Prof. Zhiyu Li in our lab [25]. GL-V9 was dissolved in dimethyl sulfoxide (DMSO, Sigma-Aldrich, St. Louis, MO, USA), made into the parent solution with the concentration of 0.1 M, and then stored at − 80 °C. The final concentration is 2.5, 5, 10, 15 and 20 μM, diluted in Dulbecco's modified Eagle medium (DMEM; GIBCO, Carlsbad, CA, USA).

Paclitaxel injection was purchased from Haikou Pharmaceutical Factory. Dorsomorphin Dihydrochloride (Compound C, C_24_H_27_ClN_5_O, MW 472.41 and purity 99.73%) was purchased from MedChem Express and dissolved in DMSO to 5 mM. N-acetyl-L-cysteine, which was from Beyotime Biotechnology, was dissolved in water to 0.5 M and was diluted with DMEM to its final concentration. Etomoxir (C_15_H_18_ClNaO_4,_ MW 320.74 and purity 98.63%) was from MedChem Express and dissolved in water to 10 mM. Dehydroepiandrosterone (DHEA, C_19_H_28_O_2_, MW 288.42) was from Solarbio Life Sciences and dissolved in DMSO to 1 M.

### 2.2 Cell culture

Breast cancer cells MDA-MB-231 were purchased from Cell Bank of Shanghai Institute of Biochemistry & Cell Biology, Chinese Academy of Sciences (Shanghai, China). Cells were cultured in DMEM with 10% heat-inactivated fetal bovine serum (Sijiqing, Hangzhou, China), 100 U/ml of penicillin and 100 μg of streptomycin at 37 °C with the CO_2_ concentration 5%. The anchorage-independent growth of MDA-MB-231 cells was performed in poly-2-hydroxyethylmethacrylate (poly-HEMA)-coated tissue-culture dishes. PolyHEMA powder was purchased from St. Louis, MO and dissolved to 12 mg/ml in 95% ethanol. The tissue-culture dishes were coated by polyHEMA-95% ethanol solution and dried at room temperature overnight and sterilized.

### 2.3 Cell morphological assessment

MDA-MB-231 cells were cultured in normal and non-adhesive poly-HEMA-treated Petri dishes. After 24, 36 and 48 h, cells were observed and photographed using the inverted light microscope.

### 2.4 Animal model

Female athymic BALB/c nude mice (6 weeks old) weighing 18–22 g were purchased from the Academy of Military Medical Sciences of the Chinese People's Liberation Army (Certificate No. SCXK-(Army) 2007–004). Animals were maintained at 23 °C with 55–65% humidity in IVC cages, with proper light, food and water supply. 2 × 10^6^ of MDA-MB-231 cells were injected into the tail vein of per female nude mice. After 7 days, the nude mice were separated by weight into the following groups (with 10 mice per group): (1) saline control group as negative control, (2) 10 mg/kg paclitaxel group as positive control, (3) GL-V9 7.5 mg/kg and (4) GL-V9 15 mg/kg (GL-V9 was prepared into lyophilized powder in the lab and dissolved in saline; paclitaxel injection were also diluted by saline.). Treatments were done by intravenous injection for once every three days. At the end of four weeks, mice were sacrificed and the lungs were removed and monitored. Lungs were fixed in Bouin's solution for 12 h and washed with 70% ethanol before taking photos. Meanwhile, animals were taken good care of according to Guide for the Care and Use of Laboratory Animals published by the National Institute of Health, USA.

### 2.5 Annexin V/PI staining assay

MDA-MB-231 cells were collected and stained with Annexin V/PI Cell Apoptosis Detection Kit (Vazyme Biotec, Nanjing, China), according to the protocols. Data acquisition and analysis were performed by Becton-Dickinson FACS Calibur flow cytometry and CellQuest software. The cells stained by neither Annexin V nor PI were regarded as survival.

### 2.6 ROS level determination

MDA-MB-231 cells were collected and stained with ROS Assay Kit (Beyotime Biotechnology, Nanjing, China), in accordance with the instructions. After collection, cells were incubated with the dye of DCFH-DA, which was attenuated with serum-free DMEM at a proportion of 1:1000, for 20 min at 37 °C in the dark. Data acquisition and analysis were performed by Becton-Dickinson FACS Calibur flow cytometry and CellQuest software.

### 2.7 Small interfering RNA (siRNA) transient transfection

G6PD siRNA was purchased from OriGene (OriGene Technologies, Inc., Rockville, MD, USA). The siRNAs targeting G6PD was delivered by a lipid-based method using Lipofectamine 2000 (Invitrogen Life Technologies, Grand Island, NY, USA) at a final siRNA concentration of 30 μM according to previous researches [26]. After formation of the siRNA–liposome complexes, the mixture was added to breast cancer cells for 4 h.

### 2.8 Western blotting assay

MDA-MB-231 cells were cultured or administrated for 36 h and then lyzed using lysis buffer and the total protein samples were isolated and eluted with SDS buffer, separated by SDS-polyacrylamide gels, and electroblotted onto PVDF membranes. The specific protein bands were detected with Odyssey Scanning System (LI-COE Inc., Superior St., Lincoln, NE) according to the previous report [27]. The following are the origins of the primary antibodies and the corresponding diluting proportions. (1) G6PD from Abclonal Technology, 1:2000, was used to determine the level of PPP; (2) β-actin from Santa Cruz Biotechnology, 1:2000, was used as internal reference; (3) p-ACC from Cell Signaling Technology, 1:1000, was used to determine the phosphorylation of ACC and the activation of AMPK; (4) CPT1A from Abclonal Technology, 1:2000, was used to determine the level of FAO; (5) PDH from Cell Signaling Technology, 1:1000, was used to determine the level of acetyl-CoA produced from glucose; (6) LDH from Abclonal Technology, 1:2000, was used to determine the level of glycolysis; (7) AMPK from Abclonal Technology, 1:2000, was used to determine the expression level of AMPK and (8) p-AMPK from Abclonal Technology, 1:2000, was used to determine the level of AMPK activation. Western blot assays for each protein were performed at least three times

### 2.9 Glucose uptake and lactic acid production determining

Cells and the medium were isolated and the medium was collected to determine the glucose uptake and lactic production level. The two detections were performed by the Amplex Red Glucose Assay Kit (Invitrogen, Eugene, OR) and Lactic Acid Production Detection Kit (KeyGen, Nanjing, China) respectively according to the instructions. Finally samples were detected by a spectrophotometer (Thermo, Waltham, MA). The absorbance for lactic acid production assay was at 570 nm and the fluorescence for glucose uptake assay was read at Ex./Em. = 530 nm/590 nm. The absorbance was normalized as follows: OD_normalized_ =OD_measured_ / living cell number_treated_ × living cell number_control_. The living cells were counted by trypan blue staining of collected cells.

### 2.10 ATP production assay

The intra-cellular ATP level was measured by ATP Assay Kit from Beyotime Biotechnology. MDA-MB-231 cells were collected and then lyzed by ATP releasing buffer on ice. According to the protocols, the standard curve of the absorbance to ATP concentrations was measured. We may then determine the ATP concentration using luminometer Orion II (Berthold DS, Bleichstr, Pforzheim, Germany).

### 2.11 Acetyl-CoA production assay

The method of the measurement of acetyl-CoA level was spectrophotometry, using acetyl-CoA Assay Kit from Solarbio Life Sciences. MDA-MB-231 cells were firstly harvested and lyzed using lysis buffer in the condition of ultrasound on ice. Then the supernatant was collected to determine the content of acetyl-CoA according to the instructions using a spectrophotometer (Thermo, Waltham, MA).

### 2.12 NADPH production assay

NADP/NADPH Quantification Kit was purchased from Sigma-Aldrich. According to the protocols, extraction buffer was added to MDA-MB-231 cells for acquiring NADP and NADPH. To detect NADPH only, NADP should be decomposed by aliquoting the extracted samples and heating them to 60 °C for 30 min in a water bath or a heating block. After a series of biochemistry reactions at room temperature for 1–4 h, the absorbance was measured at 450 nm using a spectrophotometer (Thermo, Waltham, MA).

### 2.13 Cell viability inhibition assay

The viability of MDA-MB-231 cells was measured using 3-[4, 5-dimethylthiazol-2-yl]− 2, 5-diphenyltetrazolium bromide (MTT) assay. Cells were cultured in polyHEMA-coated 6-well plate and cultured under corresponding conditions. The formazan was dissolved in DMSO and the absorbance was measured spectrophotometrically at 570 nm by the Universal Microplate Reader EL800 (BIO-TEK instruments, Inc., Vermont, MA). The inhibition ratio was calculated as the following formula:

$$\left(A_{\mathrm{control}}{-A}_{\mathrm{treated}}\right){\;/A}_{\mathrm{control}}\times \mathrm{100}\%$$

### 2.14 Plasmid extraction and transient transfection

The G6PD plasmid was designed and synthesized by Vigene Biosciences and the sequence was 5′-GAGGACTAGTTACTGTAATAGTAATCAATTACGGGG, 3′-CCTCTACAAATGTGGTATGGC. EndoFree Plasmid Midi Kit from CWBIO was chosen for the extraction of G6PD plasmid. After extraction, G6PD plasmid was stored at − 80 °C. For transfection, cells were seeded in PolyHEMA-treated 6-well plates at 65% confluency at first. Then, the plasmid DNA (1 μg) was introduced into the cells using Lipofectamine 2000 (Invitrogen Life Technologies, Grand Island, NY, USA) according to the manufacturer's recommendations.

### 2.15 Real-time PCR analysis

Total RNA was extracted using TriPure Isolation Reagent (Roche Diagnostics, Mannheim, Germany). One microgram of total RNA was used to transcribe the first strand cDNA with SuperScript II reverse transcriptase (Invitrogen). Real-time PCR was completed on an ABI PRISM Sequence Detector 7500 (PerkinElmer, Branchburg, NJ, USA) using Sequence Detector version 1.7 software (Applied Biosystems, Foster City, CA, USA). SYBR Green PCR Master Mix was purchased from Applied Biosystems. Forward and reverse primers for targeted mRNA were designed and purchased from TAKARA BiotechnologyCo., Ltd. (Dalian, China). The primer sets used in the PCR assay were as follows;

G6PD-sense: 5′-CGAGGCCGTCACCAAGAAC;

G6PD-antisense: 5′-GTAGTGGTCGATGCGGTAGA;

GAPDH-sense: 5′-GAAGATGGTGATGGGATTTC

GAPDH-antisense: 5′-GAAGGTGAAGGTCGGAGT

Fold change of mRNA level was calculated as follows. After completion of the PCR, the baselines and thresholds were set for both samples and internal GAPDH control.

### 2.16 Immunohistochemistry

The lungs were removed and stored in formalin and then sent to MyBioScience for the make of tissue slices. The paraffin embedded slices were firstly dipped in xylene, ethanol and water respectively for the removal of paraffin at 60 °C. The expression of AMPK, p-ACC, CPT1A and G6PD in the lungs of the nude mice was determined using the primary antibodies mentioned above and the diluting proportion was 1:500. In addition to this, all other reagents used in the experiment were supplied by Maixin-Bio Co. (Fuzhou, China).

### 2.17 Statistical

Data were presented as mean ± SD from triplicate parallel experiments unless otherwise indicated. Statistical analyses were performed using one-way ANOVA.

## 3 Results

### 3.1 Human breast cancer MDA-MB-231 cells own the ability of anchorage-independent growth

**Fig. 1:**
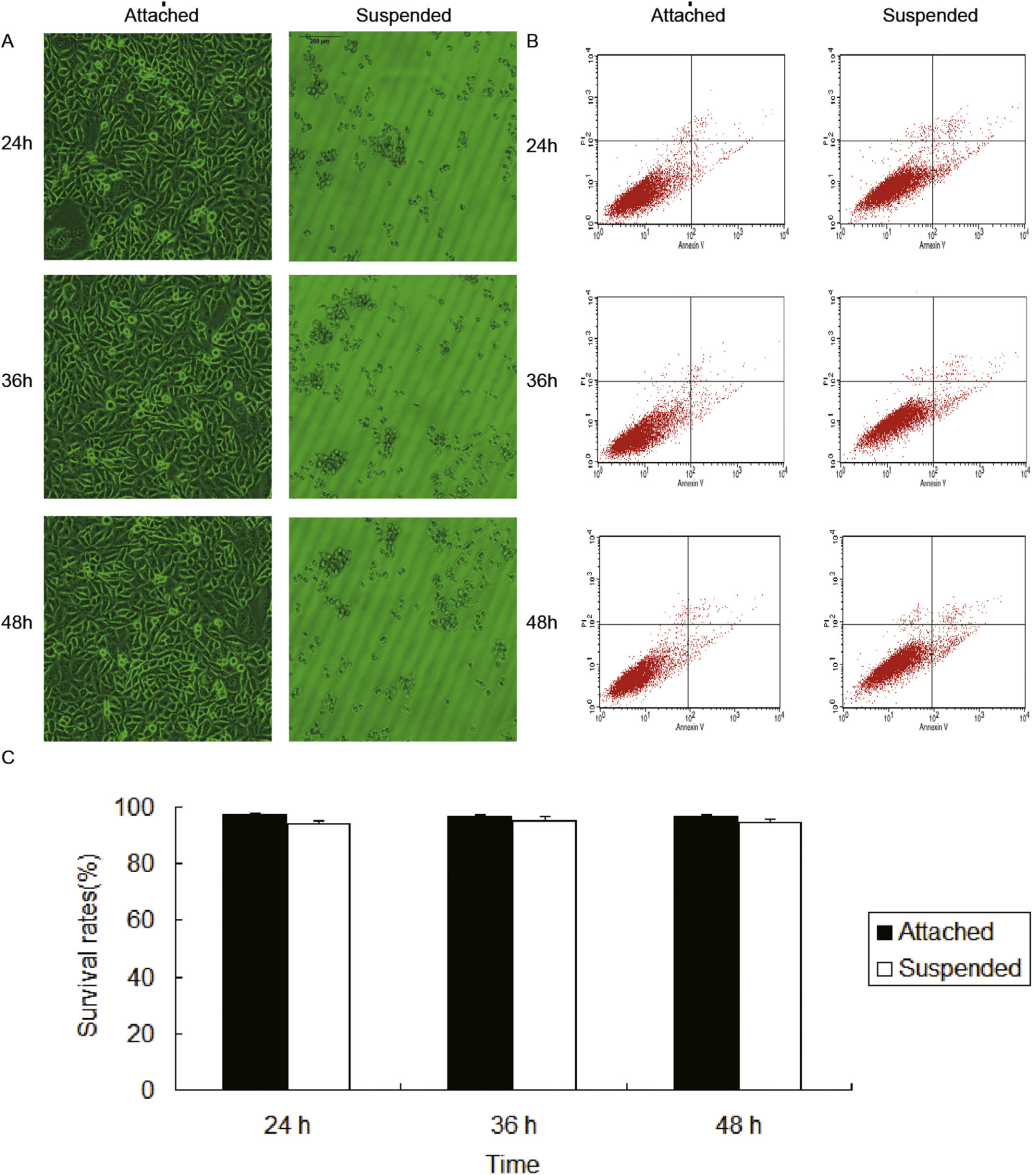
Anchorage independent growth in 3D cultures of MDA-MB-231 cells. MDA-MB-231 cells were cultured in regular dishes (attached, 2D culture), or in poly-HEMA coated dishes (suspended, 3D culture) for various times. (A) Morphology of MDA-MB-231 cells (400 ×). (B) Cell survival was determined by Annexin V/PI staining assay. (C) Quantization of cell survival rates.

In order to examine the ability of anchorage-independent growth, human breast cancer cell line MDA-MB-231 cells were cultured in non-adhesive poly-HEMA-treated Petri dishes. The cells were incubated for 24 h, 36 h and 48 h respectively, and then the level of detachment-induced cell death was determined using Annexin V/PI staining. As shown in Fig. 1A, cells seeded in normal tissue-culture dishes (2D architecture) were well-adhered and exhibited spindle-shaped morphology. In contrast, MDA-MB-231 cells on polyHEMA-treated dishes (3D architecture) formed large aggregates and suspended in culture media. The Annexin V/PI staining assay in Fig. 1B showed that detachment from the extra-cellular matrix didn’t cause obvious cell death of MDA-MB-231. The survival percentage of the attached cells at 24 h, 36 h and 48 h were 97.37%, 96.57% and 97.03%, and for the detached cells, were 94.19%, 95.36% and 94.45%, respectively (Fig. 1C). These results indicated that MDA-MB-231 cells exhibited the ability of anchorage-independent growth.

### 3.2 G6PD is a key controller of intra-cellular ROS level in MDA-MB-231 cells

**Fig. 2:**
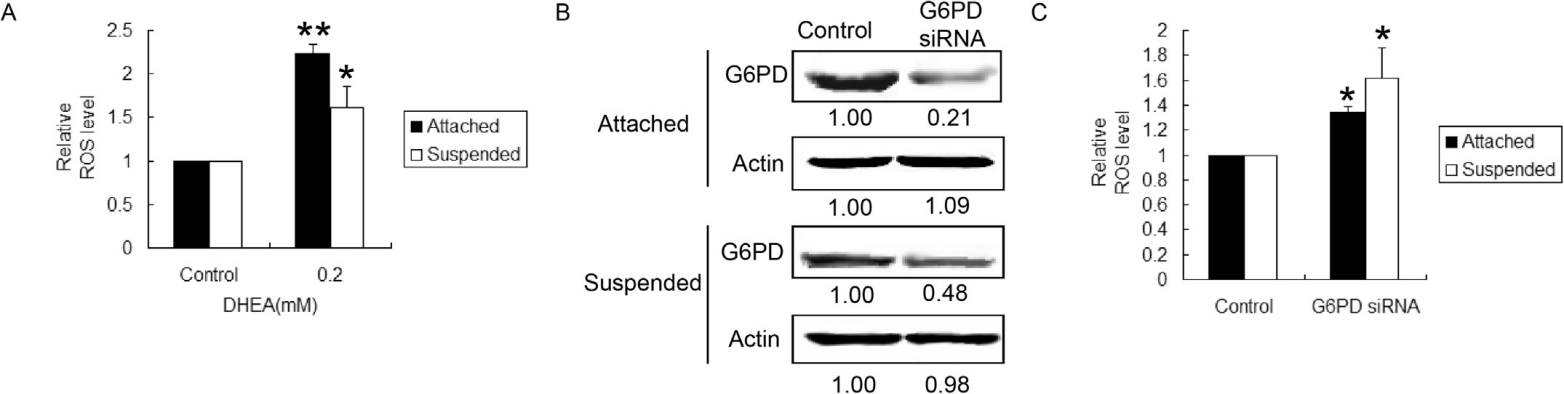
G6PD controls intra-cellular ROS level in MDA-MB-231 cells**.** MDA-MB-231 cells were cultured in regular dishes (attached, 2D culture), or in poly-HEMA coated dishes (suspended, 3D culture). (A) ROS level was determined after MDA-MB-231 cells were administrated by 0.2 mM DHEA for 36 h. (B) The transfection efficacy of G6PD siRNA. (C) ROS level was determined after cell were transfected with G6PD siRNA. *p < 0.05 and **p < 0.01 compared with control. Bars. SD.

It has been suggested that a significant advantage of aerobic glycolysis is the high flux of substrate to biosynthetic pathways such as PPP. In the oxidative branch of PPP, glucose-6-phosphate dehydrogenase (G6PD) catalyzes the rate-limiting step, which seems to be critical in controlling the intra-cellular ROS level. Therefore, we investigated the role of G6PD, which was the rate-limiting enzyme of oxidative branch of PPP, in the redox balance of MDA-MB-231 cells. Cells were cultured in normal or poly-HEMA-treated Petri dishes, respectively, and administrated with the G6PD inhibitor dehydroepiandrosterone (DHEA) for 36 h. As shown in Fig. 2A, DHEA led to a strong increase in ROS level both in attached and suspended cells. Then we transfected the two kind of cells with siRNA targeted G6PD (Fig. 2B). Knockdown of G6PD concomitantly raised the intra-cellular ROS level as well (Fig. 2C). These results indicated that G6PD is a key controller of intra-cellular ROS level in MDA-MB-231 cells.

### 3.3 FAO significantly contributes to ATP production in matrix-detached MDA-MB-231 cells

**Fig. 3:**
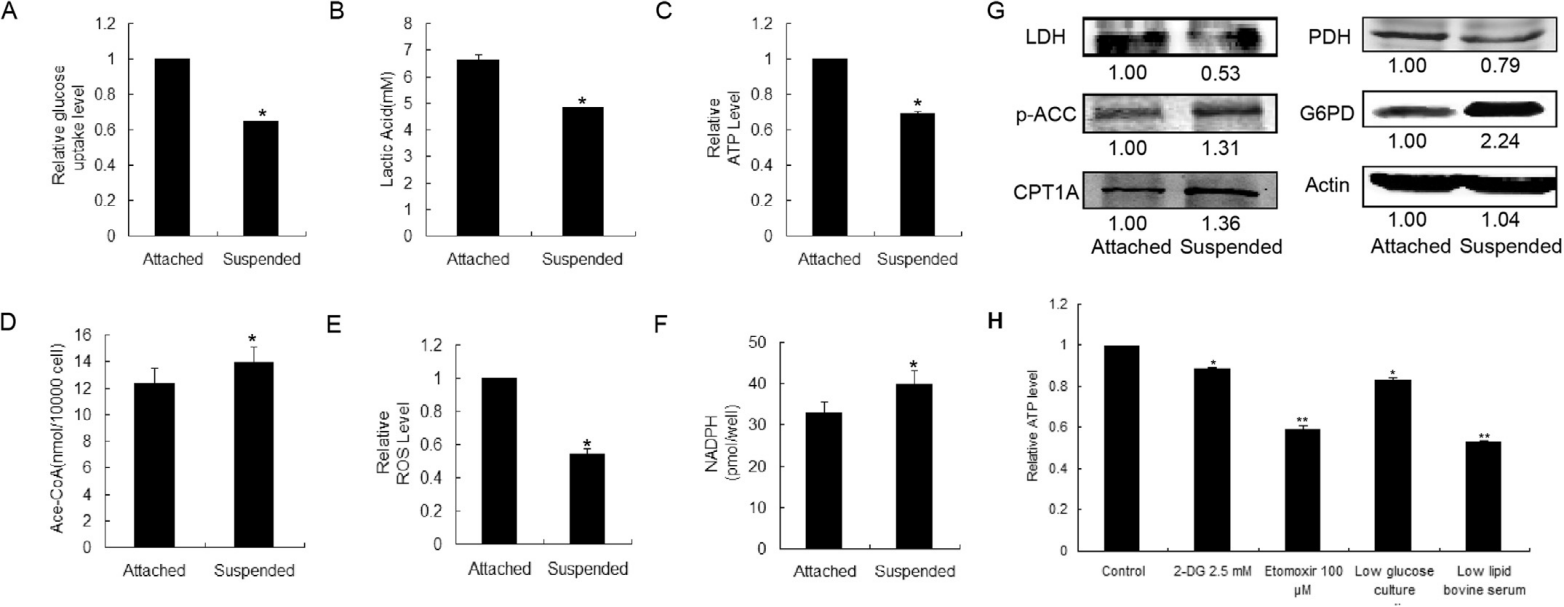
The glycolipid metabolism of MDA-MB-231 cells after detached cultured. MDA-MB-231 cells were cultured in regular dishes (attached, 2D culture), or in poly-HEMA coated dishes (suspended, 3D culture) for 36 h. (A) The glucose uptake, (B) the lactic acid production, (C) the ATP generation, (D) the acetyl-CoA production, and (E) the relative ROS level of cells in 2D and 3D cultures were measured respectively. (G) The total proteins were extracted in 2D and 3D cultures. Western blotting assay was carried out to determine the expression of LDH, p-ACC, CPT1A, PDH and G6PD, with actin as the internal control. (H) ATP generation of MDA-MB-231 cells was detected. Detached MDA-MB-231 cells were treated with 2.5 mM of 2-deoxy-glucose or 100 μM of etomoxir for 36 h. Or cells were cultured with, low glucose culture medium or low lipid bovine serum. *p < 0.05 and **p < 0.01 compared with control. Bars. SD.

Previous research has revealed that extra-cellular matrix (ECM) can regulate cellular metabolism, and fatty acid oxidation (FAO) plays an important role in ATP supply in the anchorage-independent growth of normal mammary epithelial cells MCF-10A [18]. Whether the similar phenomenon can be observed in human breast cancer MDA-MB-231 cells? We firstly detected the changes of glycolysis after MDA-MB-231 cell were suspended. As shown in Fig. 3A, B, C and G, detached culture caused substantial reduction in glucose uptake, lactic acid production and ATP production, and the protein expression of lactate dehydrogenase (LDH) was down-regulated, suggesting that after detached growth the glycolysis of MDA-MB-231 cells was weakened. Meanwhile, phospho-acetyl-CoA carboxylase (p-ACC) and carnitine palmitoyltransferase I (CPT1A)， two key enzymes in FAO, were significantly up-regulated after cells were suspended (Fig. 3G), indicating that detachment might lead to an increase in FAO. FAO is a metabolic process whereby fatty acids are broken down into acetyl-CoA, which is the product of glycolysis as well, and used for tricarboxylic acid cycle (TCA). Detachment from extra-cellular matrix caused an increase of acetyl-CoA level (Fig. 3D). During glycolysis, pyruvate is converted into acetyl-CoA by pyruvate dehydrogenase (PDH). Interestingly, PDH level was down-regulated after suspension (Fig. 3G). Moreover, we assayed the contribution of glycolysis and FAO to ATP generation in detached MDA-MB-231 cells. MDA-MB-231 cells were treated with 2.5 mM of 2-deoxy-D-glucose (2-DG, inhibitor of HKII, rate-limiting enzyme of glycolysis) or 100 μM of etomoxir (inhibitor of CPT1, rate-limiting enzyme of FAO), respectively. 2-DG lowered 12% of ATP production whereas etomoxir lowered 40.9% (Fig. 3H). Meantime, detached MDA-MB-231cells were cultured with low glucose culture medium or low lipid bovine serum. The ATP production was lowered to 83.2% and 53.0% respectively. These results suggested that it was FAO, instead of glycolysis, became the dominate source of acetyl-CoA in suspended cells and contributed more to the ATP production in MDA-MB-231 cells during anchorage-independent growth. Thus the energy metabolism pathways tend to change from glycolysis to FAO in MDA-MB-231 cells during anchorage-independent growth.

It was reported that the predominant energy metabolism in human breast cancer cell were both glycolysis and OXPHOS [28]. Thus, oxygen-dependent process containing electron transport chain would generate amount of ROS, which might induce cell death if it exceeds a certain threshold. On the other hand, detachment from ECM will lead to an increase in ROS level [18]. Unexpectedly, after cultured for 36 h in detached-dishes, MDA-MB-231 cells exhibited lower level of intra-cellular ROS levels (Fig. 3E). So we speculated if there are some adaptive changes that can keep the redox balance. As shown in Fig. 3G, after the suspended cells were cultured for 36 h, the protein expression of G6PD was significantly up-regulated, accompanied by increased NADPH level, which was one of the products of PPP (Fig. 3F).

To sum up, these results demonstrated that once breast cancer cell were detached from ECM, glucose tended to enter PPP, instead of glycolysis to keep the redox balance; whereas FAO compensated for the loss of glycolysis and contributed to ATP supply. These conclusions provide a new insight for inhibiting the anchorage-independent growth of metastatic breast cancer through modulating the metabolism-coupled redox balance.

### 3.4 GL-V9 up-regulates intra-cellular ROS level, resulting in the inhibition of anchorage-independent growth in MDA-MB-231 cells

**Fig. 4:**
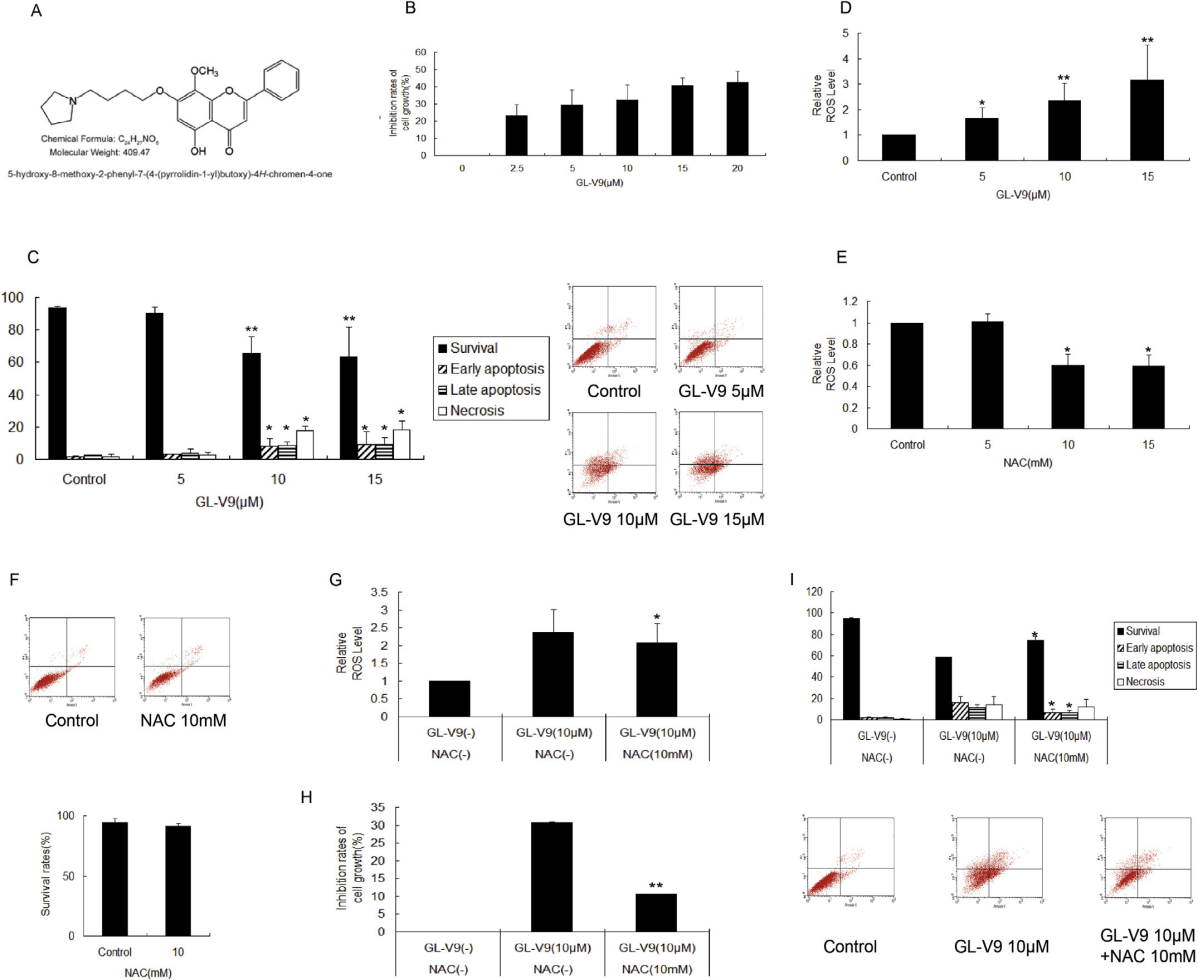
GL-V9 increases the ROS level and inhibits anchorage-independent growth of MDA-MB-231 cells. (A) Chemical structure of GL-V9. (B-D) MDA-MB-231 cells were cultured in poly-HEMA coated dishes and treated with GL-V9 for 36 h. (B) Cell viability was determined by MTT assay. (C) Cell survival was determined by Annexin V/PI staining assay and the survival rates were quantified. (D) ROS level was determined using flow cytometry. (E, F) MDA-MB-231 cells were cultured in poly-HEMA coated dishes and treated with NAC for 36 h. The ROS level (E) and cell survival (F) were measured. (G-I) MDA-MB-231 cells were cultured in poly-HEMA coated dishes and treated with 10 µM GL-V9 in the present of 10 mM NAC for 36 h. The relative ROS level (G), the inhibition rates of cell growth (H), and the survival rates (I) were determined, respectively. *p < 0.05 and **p < 0.01 compared with control (C-E) or the GL-V9 treated without NAC treatment group (G-I). Bars. SD.

GL-V9 was reported to be a potential candidate of anti-metastasis drug (the chemical structure was shown in Fig. 4A). Effects of GL-V9 on the anchorage-independent growth of MDA-MB-231 cells were detected. MDA-MB-231 cells were cultured on polyHEMA-treated tissue-dishes (3D architecture) for 36 h. MTT assay showed that GL-V9 inhibited the growth of suspended MDA-MB-231 cells in a concentration-dependent manner (Fig. 4B). Treatment by Annexin V/PI staining assay, anoikis of detached cells in a concentration-dependent manner was observed upon the treatment of GL-V9 for 36 h, significantly inhibited the survival of MDA-MB-231 cells (Fig. 4C). Besides, the main effect of lower concentration GL-V9 in detached MDA-MB-231 cells was anoikis induction, and along with the increase of GL-V9 concentration, necrosis became the dominated effect. In previous studies, GL-V9 could trigger mitochondrial mediated apoptosis and reverse hypoxia–drug resistance in human hepatocellular carcinoma, which were both involved in the regulation of redox homeostasis [29], [30]. Here, we observed that GL-V9 effectively increased the level of intra-cellular ROS in suspended MDA-MB-231 cells (Fig. 4D). In order to ensure whether the elevated-ROS contributed to GL-V9-induced inhibition of the anchorage-independent growth in MDA-MB-231 cells, we administrated cells with GL-V9 in the presence of an ROS scavenger, N-acetyl-L-cysteine (NAC). As shown in Fig. 4E and F, treatment of 10 mM NAC alone lowered the ROS level and had little effect on the survival of MDA-MB-231 cells. Then when MDA-MB-231 cells were treated with 10 μM GL-V9 and 10 mM NAC together, the increase of ROS induced by GL-V9 alone was reversed (Fig. 4G). Moreover, MTT assay and Annexin V/PI staining assay showed that the viability and the inhibition of survival in MDA-MB-231 cells by GL-V9 were diluted by NAC (Fig. 4H and I). These results indicated that GL-V9 can suppress the anchorage-independent growth of MDA-MB-231 cells through increasing ROS level.

### 3.5 GL-V9 increases ROS level through breaking the balance between PPP and FAO in MDA-MB-231 cells

**Fig. 5:**
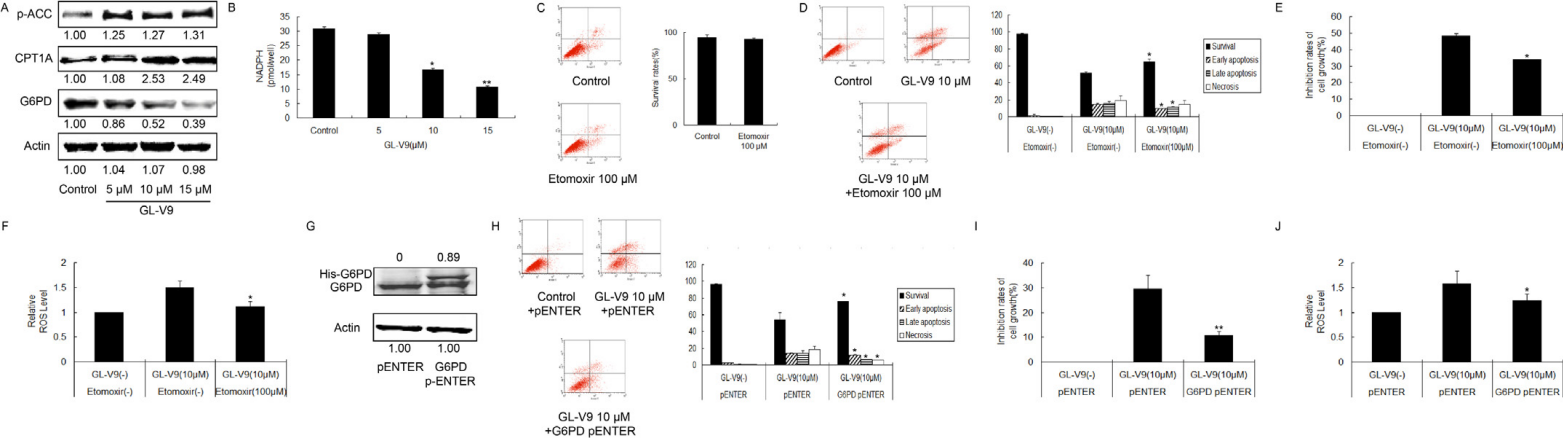
GL-V9 induced glycolipid metabolism reprogram is responsible for the inhibition of anchorage-independent growth in MDA-MB-231 cells. MDA-MB-231 cells were cultured in poly-HEMA coated dishes. (A) Western blotting assay was carried out to determine the expression of p-ACC, CPT1A and G6PD, with actin as the internal control. (B) NADPH production was determined upon GL-V9 treatment. (C) Cell survival upon 100 µM etomoxir treatment was measured and the survival rates were quantified. (D-F) MDA-MB-231 cells were treated with 10 µM in the present of 100 µM etomoxir for 36 h. (D)The survival rates, (E) the inhibition rates of cell growth, and (F) the ROS level were assayed, respectively. (G-J) MDA-MB-231 cells were cultured in poly-HEMA coated dishes, transfected with G6PD plasmids and then treated with 10 µM GL-V9 for 36 h. (G)The transfection efficacy of G6PD plasmids was detected by western blotting assay. The plasmids were His-labeled. (H) The survival rates, (I) the inhibition rates of cell growth and (J) the ROS level were determined. *p < 0.05 and **p < 0.01 compared with control or the referred group. Bars. SD.

As mentioned above, the reprogramming energy metabolism, mainly PPP and FAO, is beneficial to keep the redox balance. Therefore, we investigated whether the increased ROS level by GL-V9 in suspended breast cancer cells is associated with the imbalance between PPP and FAO. Western blot analysis showed that GL-V9 decreased the protein level of G6PD, whereas increased the protein level of p-ACC and CPT1A in suspended MDA-MB-231 cells (Fig. 5A). Meanwhile, upon GL-V9 treatment, the production of NADPH was significantly decreased (Fig. 5B). We speculated that GL-V9 increased the level of FAO and decreased the level of PPP in suspended MDA-MB-231 cells.

To determine the effect that FAO contributed to the efficacy of GL-V9, suspended MDA-MB-231 cells were treated with etomoxir, which inhibited the transport of fatty acid into mitochondria as well as the activity of CPT1A. As shown in Fig. 5C, 100 μM of etomoxir was proved to have no effect on the survival of MDA-MB-231 cells. Then, we compared the cell growth inhibitory effect between incubation with GL-V9 alone and incubation with a combination of GL-V9 and etomoxir. After MDA-MB-231 cells were administrated for 36 h, Annexin V/PI staining assay showed that 100 μM of etomoxir could partially relieve the damage of GL-V9 on MDA-MB-231 cells (Fig. 5D). Also, in MTT assay, the inhibition of GL-V9 to MDA-MB-231 cells was weakened by 100 μM of etomoxir (Fig. 5E). Moreover, co-administration lightened the increased ROS level induced by GL-V9 (Fig. 5F). These results indicated that GL-V9 increased ROS level and inhibited anchorage-independent growth partly through promoting FAO in MDA-MB-231 cells. On the other hand, we also detected the influence of GL-V9 in PPP. G6PD plasmid was transferred into suspended MDA-MB-231 cells (Fig. 5G). Annexin V/PI staining assay, MTT assay exhibited that over-expression of G6PD weakened the inhibitory effect of GL-V9 in anchorage-independent growth (Fig. 5H and I), and reduced the ROS level increased by GL-V9 (Fig. 5J). All above, these data supported that GL-V9 increased ROS level through evoking the imbalance of glycolipid metabolism, leading to the inhibition of anchorage-independent growth of MDA-MB-231 cells.

### 3.6 GL-V9 increases the expression and activity of AMPK to reprogram the glycolipid metabolism of MDA-MB-231 cells

**Fig. 6:**
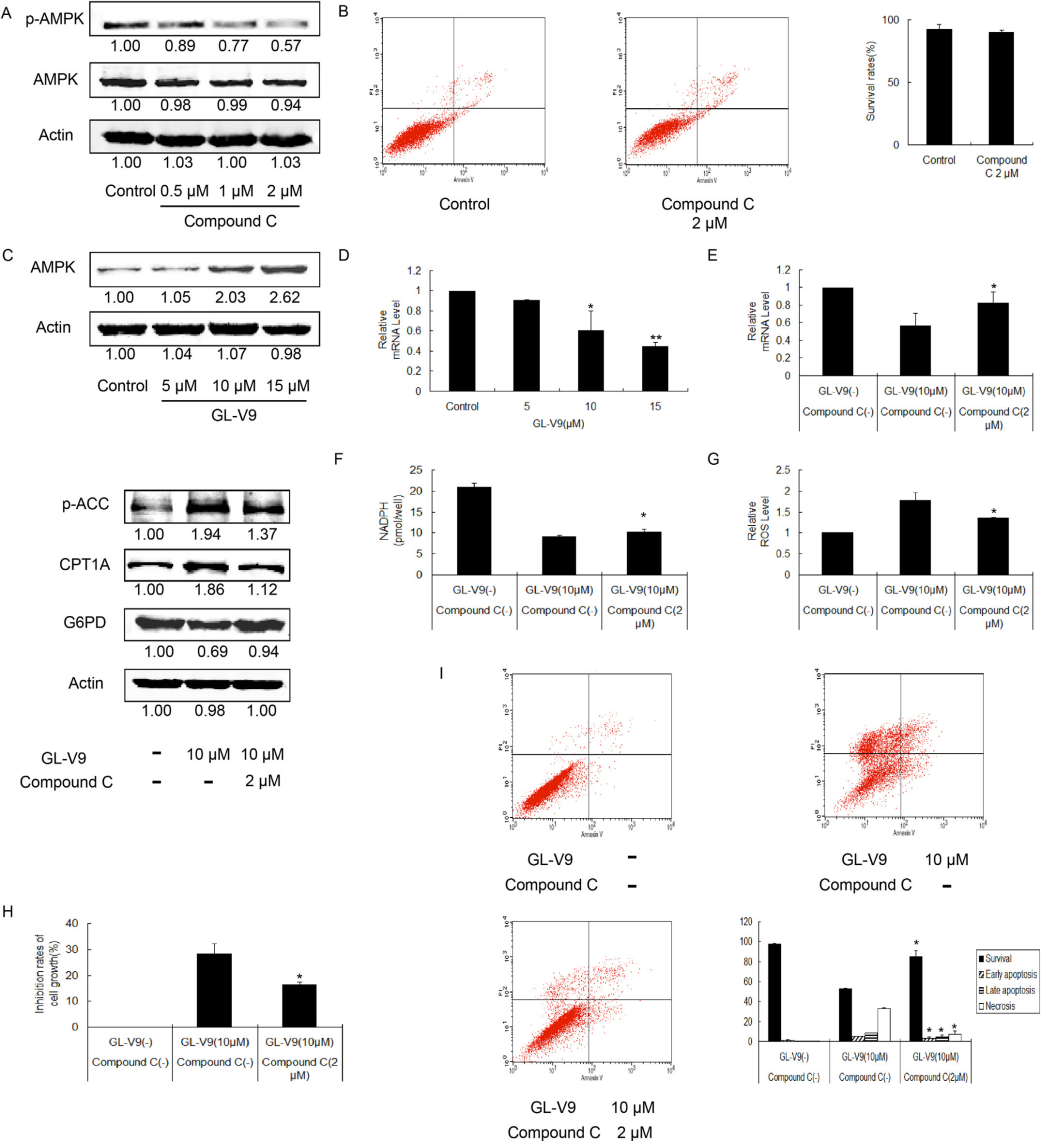
GL-V9 regulates glycolipid metabolism via increasing the expression and activity of AMPK. MDA-MB-231 cells were cultured in poly-HEMA coated dishes and treated with Compound C or GL-V9 for 36 h. (A) The expression of AMPK and p-AMPK in Compound C treated cells were assayed by Western blotting. (B) Cell survival upon Compound C treatment was determined (C) The expression of AMPK in GL-V9 treated cells, and the expression of p-ACC, CPT1A and G6PD in cells co-treated with GL-V9 and Compound C were measured. (D) The relative G6PD RNA content was analyzed after GL-V9 treatment with GAPDH as an internal control. (E-I) Cell were co-treated with 10 µM GL-V9 and 2 µM Compound C for 36 h. (E) The relative G6PD RNA content, (F) the NADPH production, (G) the relative ROS level, (H) the inhibition rates of cell growth, and (I) the survival rates were assayed, respectively. *p < 0.05 and **p < 0.01 compared with control or the referred group. Bars. SD.

A critical regulator of FAO efficacy is AMPK, the activation of which leads to the phosphorylation and inhibition of ACC. ACC can produce malonyl-CoA and inhibit FAO by decreasing CPT1A expression [31]. Herein, an inhibitor of AMPK, dorsomorphin dihydrochloride (compound C), was adopted. As shown in Fig. 6A and B, 2 μM of compound C for 36 h decreased the protein level of p-AMPK, which is the activated form of AMPK, whereas had no effect on AMPK and the survival of suspended MDA-MB-231 cells. As shown in Fig. 6C, GL-V9 treatment for 36 h increased the protein level of AMPK and CPT1A, as well as the phosphorylation of ACC. And co-administration of GL-V9 with compound C down-regulated the protein level of p-ACC and CPT1A. Thus, GL-V9 decreased ACC-CPT1A pathway by AMPK, resulting the increase of CPT1A expression and FAO level. It was reported that AMPK was an inhibitor of G6PD via inhibiting the accumulation of G6PD mRNA [32]. Therefore, we examined if GL-V9 inhibited the expression of G6PD through AMPK-G6PD signaling pathway. RT-PCR and western blot assay showed that GL-V9 down-regulated the mRNA level and protein level of G6PD, and compound C could partly reverse the effects of GL-V9 (Fig. 6D and E). Moreover, the level of the decreased NADPH caused by GL-V9 was elevated after incubation with GL-V9 and compound C together (Fig. 6F). These results demonstrated that GL-V9 inhibited PPP through the AMPK-mediated transcriptional regulation of G6PD.

Given that AMPK played an important role in the regulation of GL-V9 on PPP and FAO, we wondered whether GL-V9 inhibited the anchorage-independent growth of MDA-MB-231 cells through AMPK signaling pathway. In further studies, we found that compound C restored the increased level of ROS (Fig. 6G), and weakened the inhibition of anchorage-independent growth caused by GL-V9 (Fig. 6H and I). In conclusion, GL-V9 increased the expression and activation of AMPK, resulting in the reprogramming of FAO and PPP, the elevation of ROS level, and ultimately the inhibition of anchorage-independent growth in MDA-MB-231 cells.

### 3.7 GL-V9 suppresses the anchorage-independent growth and metastasis of MDA-MB-231 cells in vivo

**Fig. 7:**
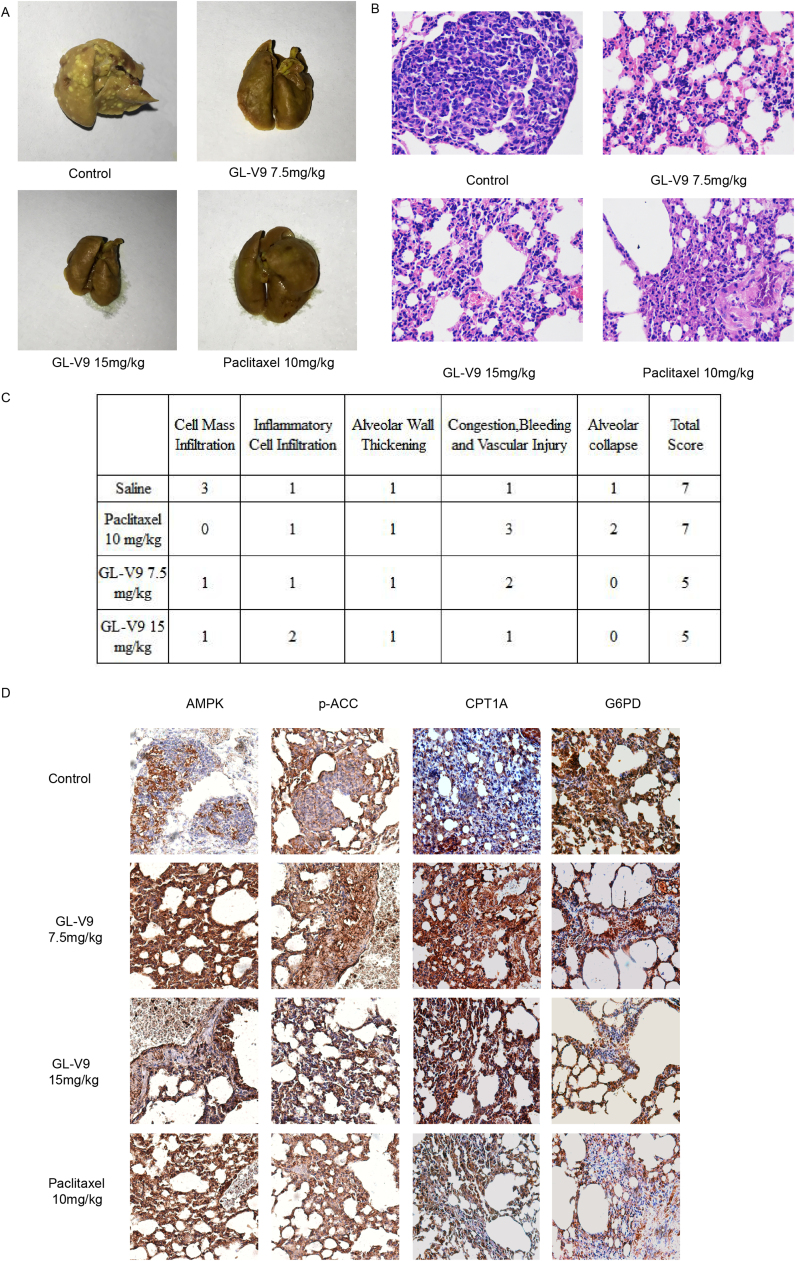
The anti-metastasis effects of GL-V9 *in vivo*. (A) The nodules formed on the lungs were photographed. (B) The cells in the nodules were detected by hematoxylin and eosin staining (400 ×). (C) Scores were carried out according to the degree of the lesion. (D) Immunohistochemical detection of AMPK, p-ACC, CPT1A and G6PD level in nodules (400 ×).

The anti-anchorage-independent growth and anti-metastatic effects of GL-V9 were further assessed with lung-metastasis model in vivo. To address the efficacy,we recorded the nodules formed on the lungs of the mice. As is shown in Fig. 7A, after 4-week administration of GL-V9 or paclitaxel, the numbers of the nodules significantly reduced. Hematoxylin and eosin staining showed that the lungs in the saline treatment group were focally infiltrated with cells, the shape of which were large, with the nucleuses round, oval or spindle, and the pathological mitotic nucleuses were visible (Fig. 7B). These cells were regarded as tumor nodules. However, after paclitaxel and GL-V9 treatment, little cell infiltration was visible (Fig. 7B). According to the degree of the lesion, scores were carried out in different sections and the total scores were the sum of each section. The higher scores were given, the more serious the lesion was (Fig. 7C). Although paclitaxel seemed to form fewer cell mass infiltrations, it caused vascular injury and alveolar collapse compared with GL-V9. Considering all these aspects, GL-V9 exhibited lower injury degrees. Immunohistochemistry for the expression of AMPK, p-ACC, CPT1A and G6PD were stained in the nodules formed in the lungs of each group. As shown in Fig. 7D, GL-V9 increased the protein level of AMPK, p-ACC and CPT1A, whereas the expression of G6PD was inhibited, which was consistent with our results in vitro. These results illustrated that GL-V9 can inhibit the anchorage-independent growth and metastasis of MDA-MB-231 cells in vivo.

## 4 Discussion

**Fig. 8:**
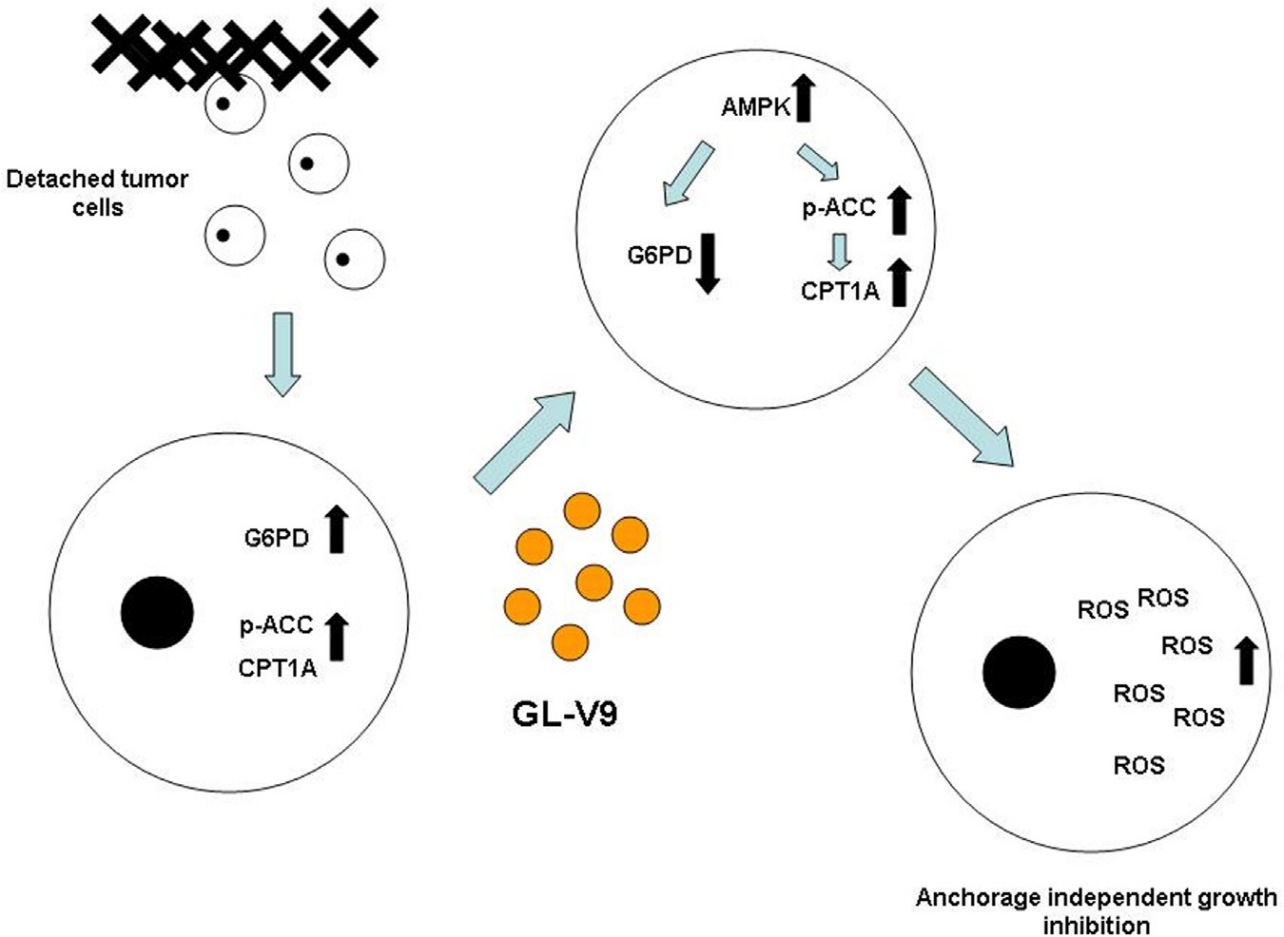
Schematic diagram of GL-V9-induced anchorage independent growth inhibition of breast cancer via reprogramming AMPK-related glycolipid. Detached breast cancer cells generated ATP through FAO, and glucose was used for PPP to scavenge excessive ROS. GL-V9 led to the decrease of G6PD as well as the increase of p-ACC and CPT1A through promoting the expression and activity of AMPK. Thus the redox homeostasis was imbalanced and resulted in the anchorage independent growth inhibition.

A considerable number of breast cancer deaths, especially TNBCs are due to metastasis. Tumor metastasis can be divided into three steps: (1) Undergoing epithelial to mesenchymal transition (EMT) and getting access to vascular and stroma; (2) Traveling through the circulatory system such as blood and lymphatic vessels; (3) Lodging in ectopic sites and forming metastatic lesion in host organs [21]. In this study, we focused on the second step of breast cancer metastasis, investigate the glycolipid metabolism reprogram of MDA-MB-231 cells in the condition of anchorage independent growth. We found that detached breast cancer cells generate ATP through FAO, whereas glucose was used for PPP to scavenge excessive ROS. Meanwhile, the flavonoid GL-V9 could lead to the decrease of G6PD as well as the increase of p-ACC and CPT1A through promoting the expression and activity of AMPK, leading to the imbalance the redox level and ultimately inhibit anchorage independent growth (Fig. 8).

The origin of ‘anoikis’ is a Greek word which means homeless [33]. The inhibition of anchorage independent growth seems to be a critical step in the inhibition of tumor metastasis. Once cancer cells get access to the extracellular stroma and enter circulatory system, it means that the tumor progression reaches the middle or advanced stage and it can be a lethal threat. Anoikis can be regarded as a special form of apoptosis despite its name and definition. Like apoptosis, anoikis can be initiated through intrinsic pathway (related to mitochondria) and extrinsic pathway (related to death receptors) [13]. Recently, another cell death manner of ECM-detached growth, named entosis, has been raised. During entosis, cell invaded into a neighbouring cell and was degradated in lysosome [34]. However, some internalized cells can remain their viability and be released ultimately. In cancer cells, the ability of anchorage independent growth is due to several mechanisms, including in the changes in integrin, the influence of EMT, pro-survival signal, metabolism reprogram and so on [11]. It has been reported that cancer cells can exploit autophagy to overcome the disadvantages in the progress of detachment [35], [36]. In our studies, we found that MDA-MB-231 cells exhibited metabolism reprogram upon suspended-culture (3D architecture), similar to normal breast epithelial MCF-10A cells. During anchorage independent growth, the glycolysis of MDA-MB-231 cells was lowered, whereas the fatty acid oxidation became the primary route of acetyl-CoA for ATP supply through OXPHOS. Notably, the glucose mostly entered PPP to resist high level of ROS, which was mainly from OXPHOS, and keep the redox balance.

AMPK plays critical roles in regulating growth and reprogramming metabolism via transcription and direct effects on metabolic enzymes, such as ACC, G6PD and HMG-CoA reductase [37]. However, the direct effects of AMPK on CPT1A were rarely reported. Most studies showed that AMPK regulated CPT1A through ACC-related mitochondrial biogenesis pathway. When ACC was phosphorylated, its activity could be inhibited, leading to a decrease in malonyl CoA accumulation and an increase in CPT1A levels [31], [38]. Our research revealed that GL-V9 inhibited the anchorage independent growth in MDA-MB-231 cells by up-regulating the expression and activity of AMPK, which reduced the PPP level and enhanced the FAO level, resulting in the imbalance of ROS eliminate and production and strong oxidative stress. Some other studies have shown that chronic stimulation (40 weeks) of AMPK activation is associated with an increase in lipogenesis. However, GL-V9 was the derivative of natural flavonoids, which usually work through multiple paths and was different with the specific AMPK activator. Latest reviews have reported that flavonoids can prevent obesity and regulate lipid metabolism, such as licorice, fisetin and so on [39], [40], [41], [42]. Besides, our previous studies showed that GL-V9 and its potentially maintain the normal body metabolic balance, inhibit lipogenesis and modify abnormal metabolism [43], [44]. It was reported that there were close relationships between AMPK, ROS and necrosis. Inhibition of AMPK activation resulted in reduction of necrosis and ROS production [45]. In our studies, GL-V9 could activate AMPK and induce necrosis under high concentration, which was in correspondence with previous report.

Intracellular lipid metabolism is regulated by a complex network. One of the hallmarks of cancer cell metabolism reprogram is increased fatty acid synthesis, which plays a central role in carcinogenesis and tumor development. Our previous research has proved that free fatty acid promoted the growth and development of human colon cancer through activating Wnt/β-catenin pathway, and natural flavonoid oroxylin A, which contains the same nuclear structure with GL-V9, could inhibit the transport and synthesis of fatty acid and suppress the colon cancer growth [43]. Recently, obesity and adverse breast cancer risk and outcome have been reviewed in a newly published article, indicating that obesity is associated with higher risk of breast cancer and worse disease outcome [3]. Therefore, focusing on obesity and lipid metabolism might provide a new insight into therapy for breast cancer. PPP branches started from glucose-6-phosphate can be divided into two branches, the oxidative branch and the non-oxidative branch. The nonoxidative branch consists of several reversible reactions and produces different glycolytic intermediates, which may convert into ribose. Compared with the nonoxidative branch, the oxidative branch produces not only intermediates for ribose synthesis but also NADPH. NADPH acts as an important intracellular ROS scavenger for it can transfer GSSH into the reduced form, GSH. Also NADPH is consumed in lipid synthesis to deliver hydrogen. G6PD catalyzes the first step, which is also the rate limit step of the oxidative branch PPP, so its expression and activity should be strengthened. GL-V9 can increase the intracellular ROS level. On one hand, the mRNA and protein expression of G6PD was suppressed. On the other hand, FAO and OXPHOS were promoted. Researches have mentioned that the decrease of PPP might increase FAO and reduced lipogenesis was indicated by reduced G6PD activities [46], [47]. Therefore more theoretical supports are needed to prove whether GL-V9 could affect the inherent connection of PPP and FAO.

To date, the mostly recommended treatment for TNBCs is surgery and radiotherapy [48], [49], [50]. Also, chemotherapy is another optional method. In clinical, taxanes combined with anthracycline drugs and platinum are mainly adopted [51], [52]. According to the researches of gemcitabine on advanced breast cancer, co-administration of gemcitabine and platinum is one of the main clinical choices in many countries [53], [54]. However, these traditional treatments may cause severe adverse effects such as high blood pressure, neutropenia and even drug resistances. According to our research, GL-V9 exhibited an effective anti-metastasis effect and no significant toxicity was investigated in accordance with our previous study [29]. This paper indicated a novel regulating mechanism of redox homeostasis involving with glycolipid metabolism, and provided a potential candidate for the therapy of metastatic breast cancer.
